# A Review of Immunological Evaluation of Patients with Recurrent Spontaneous Abortion (RSA)

**DOI:** 10.3390/ijms26020785

**Published:** 2025-01-17

**Authors:** Mihaela Andreescu, Alina Tanase, Bogdan Andreescu, Cosmin Moldovan

**Affiliations:** 1 Department of Clinical Sciences, Hematology, Faculty of Medicine, Titu Maiorescu University of Bucharest, 031593 Bucharest, Romania; 2 Department of Hematology, Colentina Clinical Hospital, 020125 Bucharest, Romania; 3 Department of Hematology, Fundeni Clinical Hospital, 020125 Bucharest, Romania; alinadanielatanase@yahoo.com; 4 Faculty of Medicine, Carol Davila University of Bucharest, 040051 Bucharest, Romania; 5 Department of Plastic Surgery, Colentina Clinical Hospital, 020125 Bucharest, Romania; doctorandreescu@yahoo.com; 6 Department of Medical Surgical Disciplines, Faculty of Medicine, Titu Maiorescu University of Bucharest, 031593 Bucharest, Romania; 7 Department of General Surgery, Witting Clinical Hospital, 010243 Bucharest, Romania

**Keywords:** natural killer cells, regulatory t cells, decidual macrophages, dendritic cells, pregnancy loss

## Abstract

In approximately half of the recurrent spontaneous abortion (RSA) cases, the underlying cause is unknown. However, most unexplained miscarriages are thought to be linked to immune dysfunction. This review summarizes the current evidence regarding the immunological evaluations of patients with RSA, with potential implications for clinical research. The immune system plays a crucial role in the successful outcome of pregnancy, as it tolerates the semi-allogeneic fetus while offering protection to both the mother and fetus from pathogens. The maternal-fetal interface is the place where the crosstalk between various immune cells such as macrophages, dendritic cells, natural killer (NK) cells, and T cells takes place. An adequate balance is required between these immune cells for pregnancy to progress. In RSA, a dysregulation between these immune players is witnessed. For example, in RSA, NK cells are not increased but also undergo a change in their activity, manifested as cytotoxic decidual NK. Similarly, regulatory T cells, which are crucial for fostering a tolerant immune environment, are decreased in RSA women. Similarly, imbalances between T-helper (Th1, Th2, Th17) cell subsets have been implicated in RSA. Furthermore, the imbalance between pro-inflammatory M1 and anti-inflammatory M2 macrophage phenotypes has been documented, with studies indicating a predominance of M1 macrophages in RSA patients. Targeting immune imbalances with therapies such as immunoglobulin administration, TNF inhibitors, and anticoagulants may improve pregnancy outcomes in women with RSA.

## 1. Introduction

Recurrent spontaneous abortion (RSA) has been defined as the occurrence of three or more consecutive pregnancies prior to the 28th week of gestation with the same sexual partner [1]. However, current evidence suggests that two consecutive miscarriages also pose a similar risk of recurrence as in cases with three miscarriages [2]. Therefore, the European Society of Human Reproduction and Embryology and the American Society for Reproductive Medicine classify patients with two or more miscarriages as RSA [2,3]. The estimated prevalence rate of RSA is less than 5% for women who have two consecutive pregnancy losses, and around 1% for 3 or more consecutive pregnancy losses [4]. In roughly half of RSA cases, the underlying causes are not identified, with around 80% of these unexplained miscarriages potentially linked to immune system factors [5]. The role of the immune system in pregnancy is multifaceted as it must not only tolerate the semi-allogeneic fetus but also ensure that the mother and fetus are protected from pathogens. A successful pregnancy requires the maternal immune system to adapt dynamically to accommodate the presence of fetal antigens inherited from the father [6]. A range of immunological processes unfold to ensure the successful outcome of pregnancy. This includes maternal-fetal tolerance, regulation of the immune cell population in decidua, and prevention of excessive inflammation [7].

The complex immunological landscape involves natural killer (NK) cells, macrophages, T cells, and dendritic cells, as well as critical mediators like cytokines and autoantibodies that influence pregnancy outcomes. These cells play a crucial role in normal pregnancy as well by establishing a balance between inflammatory response and immune tolerance [8]. For example, the role of NK cells is well established in normal pregnancy. They contribute to placental development by promoting the remodelling of spiral arteries and secreting cytokines that facilitate trophoblast invasion [9]. However, in RSA, NK cells are dysregulated and demonstrate cytotoxic activity [10]. Similar dysregulations are reported regarding other immune cells in the RSA, which result in pregnancy loss. Inflammation is a significant influence of pregnancy outcomes. In pregnancy, both inflammatory and anti-inflammatory processes prevail. Some authors suggest that inflammation might be necessary for implantation. However, the inflammatory state is later changed to a non-inflammatory state during pregnacy development [11,12]. However, there must be a balance between inflammatory and non-inflammatory mediators during pregnancy.

Reproductive immunology in pregnancy has played a pivotal role in identifying the factors contributing to pregnancy and implantation failures. However, the existing body of evidence primarily stems from smaller-scale studies. With ongoing advancements in research, there is a growing need for an updated synthesis of the current evidence to guide both researchers and clinicians. This review aims to provide a detailed examination of the current understanding of the role of the immune system in pregnancy and RSA.

## 2. Immune System and Pregnancy

The immune system is vital for the establishment and maintenance of a healthy pregnancy. The maternal immune system must undergo significant changes to ensure that the fetus, with paternal antigens, is not rejected by the body. This process involves various immune cells, cytokines, and molecular signaling pathways. All these changes take place at the maternal-fetal interface, also called decidua [13]. It is a transitory interface that only exists during pregnancy. It is derived from the uterine endometrium. Trophoblast cells are key players during pregnancy which regulate the immune system. They appear following fertilization on 4th day from a blastocyst. Their function is to supply nutrients to embryos [14]. Trophoblast regulates the immune system at the maternal site, be secreting cytokines and recruit the NK cells, monocytes, and neutrophils at the implantation site [15].

Natural killer (NK) cells, particularly decidual NK (dNK) cells, are the most abundant immune cells present in the decidua during early pregnancy. NK cells comprise 50–70% of decidua lymphocytes during the first trimester [16]. Generally, peripheral NK cells are cytotoxic; however, dNK cells at the uterine environment play a supportive role in pregnancy [17]. Vento-Tormo et al. reported that dNK1, dNK2 and dNK3 are the three main subsets of NK cells in decidua [18]. They contribute to the remodeling of spiral arteries and regulate trophoblast invasion [19]. NK cells are very low during the pre-ovulatory stage; however, with the increase in progesterone levels, the NK cell is elevated during the peripheric secretory phase. Again, NK cells are reduced at the end of menstrual cycle [12].

During pregnancy, NK cells also promote immune tolerance by suppressing Th17-mediated local inflammation [20]. They achieve this primarily through the release of IFN-γ secreted by CD56brightCD27+ subset of NK cells. However, this function of NK cells becomes compromised in inflammatory conditions. For instance, in patients with RSA, non-NK cells in decidua secrete high levels of IL-1β and IL-6, which promote the expansion and recruitment of TH17 cells. The regulatory role of NK cells can be compromised in the presence of abnormal inflammation, leading to the loss of TH17 cell control and ultimately to the loss of tolerance [20]. Furthermore, NK cells release a range of angiogenic factors that support the development of the placenta and uterine environment. Among these factors are vascular endothelial growth factor (VEGF) and angiopoietin-2, along with various cytokines and growth factors such as GM-CSF, placental growth factor (PlGF), and IFN-γ. VEGF is believed to promote localized angiogenesis as part of forming decidual and mesometrial lymphoid aggregates (MLAp) essential for sustaining pregnancy [21].

The role of T cells in pregnancy is well-established. Naïve T cells (TN) typically circulate within the bloodstream, remaining in a resting state until dendritic cells present their specific antigens in secondary lymphoid tissues. It triggers the T cells to proliferate and differentiate into effector cells performing many immunologic functions. Antigen-specific T cells that have participated in resolving an immune threat persist as memory T cells, enabling a faster response if the same antigen is encountered again. [22,23]. During pregnancy, maternal T cells recognize fetal antigens through interactions with antigen-presenting cells, and playing a crucial role in establishing immune tolerance between mother to the fetus. Tolerance involves fetal-specific T cells that help promote a state of T cell acceptance. Various T cell subsets, such as regulatory T (Treg) cells, as well as T helper (Th)-1, Th2, and Th17 cells, contribute to both activating and suppressing immune activity to maintain the tolerance [24].

The Th1/Th2 immune response model was first introduced by Mosmann and colleagues in 1986 [25]. Since then, significant advancements in immunology have revealed additional Th subtypes at the maternal-fetal interface, including Th9, Th22, and T follicular helper (TFH) cells, which play distinct roles compared to the well-known Th1, Th2, and Th17 cells in pregnancy [26,27]. In this framework, CD3+CD4+ T helper cells interact with HLA class II molecules, while CD3+CD8+ cytotoxic T cells recognize HLA class I molecules. Upon activation, T helper cells produce and release cytokines that are essential for activating cytotoxic T cells, promoting B cell antibody class switching, and enhancing the pathogen-destroying functions of phagocytic cells such as macrophages [28]. During the peri-implantation phase, the immune system tends to shift towards a Th1-dominant response, characterized by mild inflammation. Contrary to causing harm, this controlled inflammatory state supports the invasion of trophoblasts, essential for establishing early pregnancy. Following placental implantation, a noticeable shift toward a Th2-dominant response occurs, which is crucial for maintaining a healthy pregnancy. The transition helps support fetal and placental development while suppressing Th1 activity at the maternal-fetal interface to prevent excessive inflammation [29,30].

**Figure 1:**
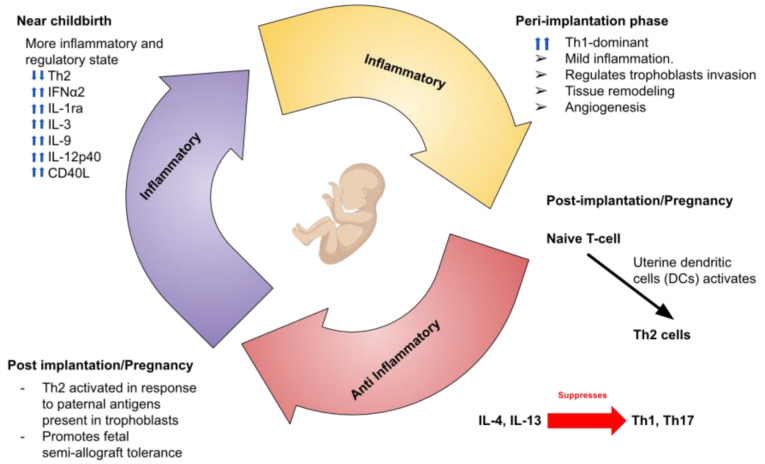
Pregnancy can be divided into 3 phases including inflammatory, anti-inflammatory, and again inflammatory stage near birth. During peri-implantation phase, inflammation is required for angiogenesis and trophoblast invasion, ensuring proper implantation. During post-implantation stage, Naïve T cells are converted to Th2 cells. Furthermore, IL-4 and IL-13 suppress Th1 and Th17, ensuring immune tolerance. Prior to birth, inflammatory stage is present.

In pregnancy, uterine dendritic cells (DCs) are pivotal in directing naive T cells to become Th2 cells [31]. At the maternal-fetal interface, Th2 cells are activated in response to paternal antigens present in trophoblasts, leading to their migration into the decidua basalis [32]. These cells become Th2 effector cells at the site and secrete Th2-associated cytokines, shifting the local milieu toward a Th2 response, which is required for tolerance at the maternal-fetal interface. The cytokines, IL-4 and IL-13, are produced by Th2 cells and suppress the development of Th1 and Th17 immune response, thus promoting fetal semi-allograft tolerance [33,34]. As pregnancy advances, there is a gradual shift in the maternal immune system away from a predominantly Th2-driven response towards a more inflammatory and regulatory state. This transition is part of the natural preparation for childbirth. Research examining serum samples from pregnant women during the second trimester found that levels of certain inflammatory markers, including IL-1β, IL-6, IL-8, IL-12p70, IL-13, IL-15, IP-10, and FLT3-ligand, increased in relation to gestational age. Meanwhile, other markers like IFNα2, IL-1ra, IL-3, IL-9, IL-12p40, and soluble CD40L showed a rise as pregnancy progressed into later trimesters [35]. This shift from Th2 immunity to a more inflammatory profile is thought to be a key factor in preparing the body for labor and delivery (Figure 1).

Regulatory T cells (Tregs) also play a pivotal role in maintaining maternal-fetal immune tolerance. Tregs are a subset of T cells that help modulate immune responses and, in general, prevent autoimmunity by suppressing the activation and proliferation of other cytotoxic immune cells that could potentially target fetal antigens. It was established that pregnancy women have high levels of FoxP3+ and Foxp3− Tregs compared to non-pregnant women [36,37]. Treg cells play a vital role in managing inflammation during the shift from a proinflammatory immune state to an anti-inflammatory decidual environment, which is essential for embryo implantation and the continuation of pregnancy [38]. Numerous studies have demonstrated that Treg cells help protect against damaging maternal immune responses toward the fetus by curbing an overactive Teff-cell response [39,40]. Insufficient numbers of Treg cells in the decidua result in an excessive Teff-mediated immune reaction, potentially causing fetal loss [38]. Therefore, a balance is required among various immune cells to develop a healthy pregnancy.

Th17 cells play a significant role due to their ability to drive or regulate inflammation, depending on the situation. This adaptability is especially important in the placenta and reproductive tract, where Th17 cells adjust their activity to support a healthy pregnancy [41]. Some studies suggested that the level of IL-17 increases during the last trimester of pregnancy [42]. Another study showed that IL-17 increases the progesterone secretion [43]. The inflammatory nature and ability to promote progesterone secretion highlight the lability of Th17 and pregnancy outcomes. However, other studies reported that the levels of Th17 do not change significantly throughout pregnancy rather Treg/Th17 ratios decrease at the end of gestation [44,45]. The pro-inflammatory nature of Th17 cells, combined with the immune-regulating role of Treg cells, creates a balance that is key to both immune tolerance and defense [46]. Similarly, a prospective study by Braga et al. reported that Treg cells are consistently reduced throughout pregnancy; Th17 levels remain constant during pregnancy [47].

Cytokines are critical mediators of immune communication and regulation during pregnancy. The balance between pro-inflammatory and anti-inflammatory cytokines are particularly important. In early stages of pregnancy, a mild pro-inflammatory environment is necessary for implantation and placental development. This process is marked by the presence of cytokines, including tumor necrosis factor-alpha (TNF-α) and interleukin-6 (IL-6), which support tissue remodeling and promote trophoblast invasion [48]. As pregnancy progresses, a shift towards an anti-inflammatory state is required to maintain fetal tolerance and prevent immune rejection. This phenomenon is supported by anti-inflammatory cytokines like interleukin-10 (IL-10) and transforming growth factor-beta (TGF-β), which help sustain the pregnancy by promoting immune tolerance and preventing harmful inflammatory responses [49].

## 3. Main Players in RSA

### 3.1. Natural Killer Cells

**Figure 2:**
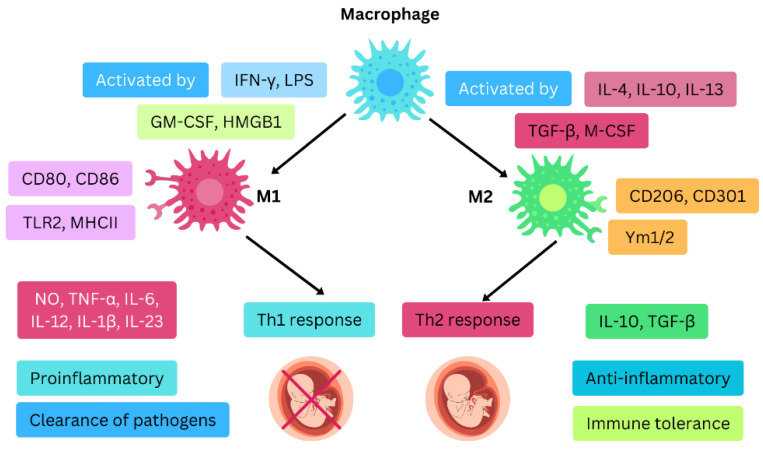
M1 macrophages are induced by INF-γ, LPS, GM-CSF, and HMGB1 whereas M2 are activated by IL-4, IL-10, IL-13, TGF-β, and M-CSF. M1 macrophages have cell markers including CD80, CD86, TLR2, MHCII whereas M2 have CD206, CD301, and Ym1/2. M1 is associated with Th1 response, including cytokine production of NO, TNF-α, IL-6, and IL-23. Furthermore, M1 produce chemokines including CXCL9, CXCL10. M2 leads to Th2 response needed for immune tolerance, with associated cytokine production (IL-10, TGF-β) and chemokine production (CCL17, CCL18). In normal pregnancy M2 phenotype is abundant whereas in RSA M1 is abundant.

During normal pregnancy, adequate balance is mandatory between the activation and inhibition of NK cells cytotoxicity which is mediated by some receptors including inhibitory and activator receptors (Figure 2). Prior studies have shown that patients with RSA have increased levels of uNK cells in the mid-secretory phase endometrium [21]. For example, Hadinedoushan et al., in their study reported that RSA patients had higher NK cell cytotoxicity within 24 h after abortion. Apart from NK cytotoxicity, they also noted elevated levels of IL-2 in RSA patients [50]. Similarly, Karami et al. found that RSA subjects have higher NK cell cytotoxicity compared to healthy controls [51]. Some authors have reported that NK cell and their activity tends to change in recurrent abortions, with primary aborters having the highest levels and NK cell activity [52].

The interactions between uNK cells and trophoblasts are crucial for the success of the pregnancy. NK identify self-cells through killer-cell immunoglobulin-like receptors (KIRs) present on their surface. They interact with their ligands which are called human leukocyte antigens (HLAs) present on self-cells. Trophblasts express HLA-C, as the only classical HLA class I molecules [53]. During pregnancy, inhibitory killer immunoglobulin-like receptors (KIRs) recognize HLA class I molecules, particularly HLA-C and HLA-G, and subsequently transmitting signals that suppress the cytotoxic activity of natural killer (NK) cells, thereby protecting self-cells from damage. In contrast, activating immunoglobulin-like receptors (KIRs) induce natural killer (NK) cell activation, promoting the destruction of target cells. The equilibrium between activating and inhibitory signals mediated by KIRs is essential for maintaining immune homeostasis during pregnancy. In the context of RSA, the KIR-HLA interaction becomes particularly important.

KIR has eight inhibitory genes and three activatory genes. Among inhibitory KIR genes, the level of *KIR3DL1* inhibitory genotype rarely found among RSA women. Ataei et al. confirmed that patients with RSA had significantly lower levels of *KIR3DL1* compared to control (82% vs. 57.5%; *p* = 0.012) [54]. Similarly, a meta-analysis by reported that *KIR3DL1* was a protective factor for RSA (*p* = 0.044; OR = 0.833) [55]. They further revealed that *KIR2DS2* and *KIR2DS3*, both activating genes were a risk factors for RSA. However, Mansour et al., Also identified low levels of *KIR2DS2* and *KIR2DL5A* in RSA compared to healthy controls in their study [56]. Study performed in Saudi Arabia which included 69 RSA women reported that *KIR3DL1*, *2DS4ins*, *2DL2*, and *KIR2DS2* were significantly low in RSA women compared to healthy controls (*p* < 0.01) [57]. Su et al. also veryfied that women who had ≥3 miscarriages had a lower frequency of KIR3DL1 compared to controls [58].

HLA-C molecules are divided into two groups based on their alleles: HLA-C1 and HLA-C2. Studies have shown that certain combinations of maternal KIR and fetal HLA-C can either promote a successful pregnancy or increase the risk of RSA. For example, women with the KIR AA genotype, which lacks activating KIRs, are at a higher risk of RSA when their fetus inherits an HLA-C2 allele from the father. Women with the KIR AA genotype has fewer activating receptors to counterbalance the strong inhibitory signals from the interaction between KIR2DL1 and HLA-C2 [59]. Furthermore, KIR AA/HLA-C2 binding leads to inadequate activation of uNK and poor placental development [60]. Table 1 shows the summary of studies regarding the role of KIR in RSA.

### 3.2. T Cells

T cells are a key component of the adaptive immune system, with various subsets of T cells playing crucial roles at the maternal–fetal interface. Tregs are a subset of CD4+ T cells characterized by the expression of the transcription factor FoxP3. The role of Tregs in preventing RSA is highlighted by studies showing that women with RSA often have reduced numbers or impaired function of Tregs. A systematic review and meta-analysis reported that women with RSA have lower levels of Tregs in peripheral blood compared to healthy controls [65]. Similarly, a study by Zeng et al. reported that patients with RSA had lower levels of peripheral CD4+CD25highCD127low/−Tregs with lower FOXP3 expression [66]. The reduction in Treg numbers may contribute to an increased risk of fetal rejection due to the lack of adequate immune regulation. Another study reported that low levels of CD4 (+) CD25 (+) Foxp3 (+) T regulatory cells can predict the risk of RSA [67]. Normally, Treg cells suppress inflammation and maternal immunity during pregnancy [68]. However, low levels of Tregs as seen in RSA can lead to a breakdown of the anti-inflammatory state which results in fetal rejection. Moreover, functional impairments in Tregs, such as decreased expression of suppressive cytokines like IL-10 and transforming growth factor-beta (TGF-β), have also been associated with RSA. A study by Farshchi et al. reported that reduced levels of TGF-β are present in the cervicovaginal fluid of patients with RSA compared to healthy controls. They further found elevated levels of IL-17 and FOXP3 mRNA in RSA patients [69].

The mechanisms through which Tregs contribute to RSA primarily involve an imbalance of the Th1/Th2/Th17/Treg cells paradigm and the abnormal proportion and activity of Tregs. Helper T cells (Th cells), particularly Th1, Th2, and Th17 subsets, play a significant role in modulating immune responses during pregnancy. The balance between these subsets is crucial for maintaining the delicate immune equilibrium required for a successful pregnancy. Dysregulation of T lymphocyte homeostasis is also involved in the etiology of RSA. Th1 cells produce pro-inflammatory cytokines such as IFN-γ and tumor TNF-α, which are involved in immune responses against infections but can also contribute to inflammation and tissue damage if not properly regulated. Th2 cells, in contrast, produce anti-inflammatory cytokines like Il-4, Il-5, and IL-13, which promote immune tolerance and protect the fetus from maternal immune attack [70]. In a healthy pregnancy, there is a preferential shift towards a Th2-dominant response to maintain an anti-inflammatory environment that favors fetal survival. In peripheral blood from patients with RSA, the balance between Th1 and Th2 cells is disrupted in favor of Th1 cells, and the ratio of Th17/Treg cells is skewed toward Th17 cells [71,72]. Elevated levels of Th1 cytokines, such as IFN-γ and TNF-α, have been observed in women with RSA [73]. Similarly, another study showed that the number of Th1 cells and the Th1/Th2 ratio were elevated in patients with RSA compared to those in normal pregnant controls [74].

### 3.3. Macrophages

During normal pregnancy, macrophages are among the most abundant immune cells in the decidua, the specialized uterine lining that forms during pregnancy to support embryo implantation and placentation These decidual macrophages play key roles in tissue remodeling, angiogenesis, apoptosis, trophoblast invasion, and suppression of excessive inflammation [75]. Decidual macrophages account for 20–30% of the leukocytes in the decidua [76,77]. Based on their cytokine secretion profiles, chemokine expression, and functional roles, decidual macrophages are categorized into two main subsets: M1 (classically activated) and M2 (alternatively activated) macrophages [78]. The M1 phenotype is associated with producing pro-inflammatory cytokines such as tumor TNF-α and IL-1β which are crucial for early pregnancy stages to support tissue remodeling and trophoblast invasion. Conversely, M2 macrophages secrete anti-inflammatory cytokines like interleukin-10 (IL-10) and transforming growth factor-beta (TGF-β), which promote immune tolerance and protect the fetus from maternal immune attack as the pregnancy progresses [79]. Macrophages are typical plastic cells which can switch phenotypes and be subject to environmental disturbances. Therefore, the dynamic shift from an M1 to an M2 macrophage phenotype is essential for the progression of a healthy pregnancy [80].

Generally, in response to infection, macrophages initially adopt a pro-inflammatory M1 phenotype to help the body fight off pathogens. After this initial defense, they gradually transition to an anti-inflammatory M2 phenotype to aid in tissue repair [81]. During the early stages of pregnancy, specifically around the time of implantation, macrophages are directed toward the M1 activation state. As trophoblasts begin to attach to the endometrium and invade the uterine tissue, these immune cells adapt to a mixed M1/M2 state [82]. This blend of pro- and anti-inflammatory macrophages continues throughout the first trimester and into the early part of the second trimester, a period during which the uterine blood vessels are being remodeled to support a healthy placental and fetal blood flow (Figure 2). Following the completion of placental development, macrophages predominantly shift to the M2 phenotype. This shift helps prevent an immune response against the fetus, promoting fetal growth until birth [83].

As labor approaches, the immune environment becomes pro-inflammatory again, characterized by an increase in M1 macrophages within the uterus [83]. The ability of macrophages to shift between these functional states is known as macrophage polarization [84]. This polarization occurs in response to the specific signals and conditions in their surrounding environment, allowing macrophages to perform different roles as needed throughout pregnancy [85]. The polarization of macrophages into M1 or M2 types is influenced by how they metabolize arginine, which follows two opposing pathways. M1 macrophages emerge from the iNOS pathway, which converts arginine into citrulline and nitric oxide (NO). In contrast, M2 macrophages originate from the arginase pathway, leading to the production of ornithine and urea from arginine [86].

In the early stages of pregnancy, M1 macrophages play a role in clearing apoptotic cells, preventing infections, and supporting initial tissue remodeling. However, as the pregnancy advances, there is a necessary shift towards an M2-dominant environment that promotes tissue repair, angiogenesis, and immune tolerance. This M2 polarization is critical for preventing an overactive immune response that could lead to fetal rejection [78]. Dysregulation of this balance between M1 and M2 macrophages has been associated with RSA. Tsao et al., in their study, reported that patients with RSA had abundant M1 macrophages whereas individuals with normal pregnancy had higher levels of M2 [87]. Severe other studies have also reported that disturbance in the M1/M2 macrophage balance is associated with adverse pregnancy outcomes [88,89].

Macrophages also play a direct role in trophoblast invasion and placental development. They contribute to this process by producing enzymes like matrix metalloproteinases (MMPs), which degrade the extracellular matrix and facilitate trophoblast invasion. Additionally, macrophages secrete angiogenic factors, including vascular endothelial growth factor (VEGF) and placental growth factor (PlGF), which promote the formation of new blood vessels and ensure adequate blood supply to the developing placenta. In cases of RSA, impaired macrophage function may lead to inadequate trophoblast invasion, poor placental vascularization, and, ultimately, pregnancy failure [90].

### 3.4. Dendritic Cells

During normal pregnancy, dendritic cells (DCs) are present in the decidua, the specialized endometrium that lines the uterus during gestation. Here, they play a crucial role in shaping the local immune environment to support fetal development. DCs are crucial for initiating the activation of naïve T cells and guiding their differentiation into Th1, Th2, Th17, and Treg subsets [91]. Dendritic cells in the decidua are generally in an immature state, characterized by low expression of costimulatory molecules and a reduced capacity to stimulate T cells, which promotes immune tolerance. DCs are also involved in the production of cytokines, such as interleukin-10 (IL-10) and transforming growth factor-beta (TGF-β), which help to create an anti-inflammatory environment conducive to maintaining pregnancy. Qian et al. reported that RSA patients had increased levels of mature DCs and a decrease in the number of immature DCs in decidua [92].

## 4. Cytokines and Chemokines in RSA

Cytokines and chemokines are critical in mediating immune responses during pregnancy. An imbalance in pro-inflammatory (e.g., TNF-α, IL-6) and anti-inflammatory (e.g., IL-10) cytokines has been observed in women with RSA. Elevated levels of pro-inflammatory cytokines can create an environment that is hostile to fetal development, while adequate levels of anti-inflammatory cytokines are necessary for successful implantation and maintenance of pregnancy.

Two types of NK cells, NK1 and NK2, present at the feto-maternal interface. NK1 cells secrete interferon-gamma (IFN-γ) and tumor necrosis factor-alpha (TNF-α), while NK2 cells release interleukins such as IL-4, IL-5, IL-10, and IL-13 [93]. Among these cytokines, IL-10 is particularly important for successful pregnancy outcomes, while IFN-γ and TNF-α contribute to angiogenesis and the growth of trophoblast cells. Deviations in cytokine levels have been associated with adverse pregnancy outcomes [94]. Dong et al. found that the levels of Th1/Th2 and dNK1/dNK2 were elevated in women with a history of recurrent miscarriage compared to a control group [95].

One of the most studied cytokines in the context of RSA is TNF-α, a potent pro-inflammatory cytokine produced primarily by macrophages and T cells. TNF-α plays a role in the early stages of pregnancy by promoting decidualization, vascular remodeling, and trophoblast invasion. However, elevated levels of TNF-α have been consistently reported in women with RSA [96]. Li et al. and Alkhuriji et al. reported that TNF-α is the risk factor for RSA [97,98]. Similarly, Begum et al. found elevated levels of serum TNF-α levels in RSA patients [99]. Due to the role of TNF-α in RSA, Jiang et al. investigated the effect of TNF inhibitor along with immunoglobulin and heparin in RSA patients. Their findings showed that TNF inhibitors with immunoglobulin and heparin treatment improved live birth rate in RSA patients [100]. While evaluating the levels of TNF-α, it should also be kept in mind that plasma TNF-α levels rise in patients with recurrent pregnancy loss (RPL) during the first trimester, irrespective of the pregnancy outcome. Notably, TNF-α levels are often elevated in cases of secondary RPL compared to primary RPL, possibly due to genetic predispositions [101]. This difference may be attributed to a prior sensitization to fetal or trophoblast antigens during an earlier pregnancy, which could trigger a humoral or cytotoxic response to these antigens in a subsequent pregnancy, thereby leading to inflammatory reactions during the early stages of pregnancy [70]. High TNF-α levels are associated with increased apoptosis in trophoblast cells, which can impair placental development and function [102].

IL-6 is another pro-inflammatory cytokine that has been implicated in RSA. While IL-6 plays a dual role in pregnancy, supporting both pro-inflammatory and anti-inflammatory processes depending on the context, its dysregulation can be harmful. Elevated IL-6 levels have been observed in women with RSA. Zhu et al., in their study, reported an increase in the IL-6 cytokines and it had a positive correlation with Th17 [103]. Similarly, Thaker et al. reported that IL-6 was positively correlated with number of recurrent abortions whereas IL-10 was negatively [104].

## 5. Autoantibodies and RSA

### 5.1. Antiphospholipid Antibodies (aPL)

Antiphospholipid antibodies are the most studied autoantibodies in the context of RSA. They are primarily associated with antiphospholipid syndrome (APS), an autoimmune disorder characterized by thrombosis and pregnancy complications. The presence of aPL, such as aCL and anti-β2GPI, leads to abnormal placental development, vascular thrombosis, and interference with trophoblast function, all of which can cause fetal demise. Several studies have confirmed the association between aPL and RSA, indicating that these antibodies contribute to pregnancy loss by activating complement pathways and inducing pro-inflammatory responses within the placenta [105,106]. Treatment with anticoagulants, such as low-dose aspirin and heparin, has been shown to improve pregnancy outcomes in women with aPL-related RSA, further underscoring the role of these antibodies in the pathology of recurrent pregnancy loss [107].

### 5.2. Antinuclear Antibodies (ANA)

Antinuclear antibodies are another class of autoantibodies frequently detected in women with RSA. ANA target various nuclear antigens, and their presence suggests an underlying autoimmune condition such as systemic lupus erythematosus (SLE). ANA can cause pregnancy loss by inducing inflammation, placental damage, and disrupting normal placental development [108]. They may also contribute to RSA by forming immune complexes that deposit in the placental vasculature, leading to impaired blood flow and nutrient exchange. Although the association between ANA and RSA is less well-defined compared to aPL, some studies have reported a higher prevalence of ANA in women with RSA, particularly in those without other identifiable causes of pregnancy loss. Shankarkumar et al., in their study reported that RSA patients had higher levels of ANA compared to controls (10% vs. 4%) [109]. Similarly, a meta-analysis by Chen et al. revealed that ANA positivity was a risk factor for RSA [110].

### 5.3. Anti-Thyroid Antibodies

Anti-thyroid antibodies, including anti-thyroperoxidase (TPO) and anti-thyroglobulin (TG) antibodies, have also been implicated in RSA. These antibodies are markers of autoimmune thyroid disease (AITD), which is associated with an increased risk of miscarriage. A meta-analysis of 22 studies reported a significant association between TPO antibodies and RSA (OR = 1.85; 95% CI, 1.38 to 2.49; *p* < 0.001) [111]. Similarly, Song et al., in their meta-analysis showed that patients with anti-TPO and/or anti-TG antibodies have a higher risk of RSA compared to those without these antibodies [112]. Liu et al. further identified that the risk of miscarriage was highest in the first trimester in women with RSA [113]. The exact mechanism by which anti-thyroid antibodies contribute to RSA is not fully understood, but it is thought to involve both direct and indirect effects. Directly, these antibodies may cross-react with placental tissues, leading to local inflammation and trophoblast dysfunction [114]. Indirectly, they may alter thyroid function, leading to hypothyroidism, which is known to adversely affect pregnancy outcomes.

### 5.4. Anti-Cardiolipin Antibodies (aCL)

Anti-cardiolipin antibodies are a subset of antiphospholipid antibodies that specifically target cardiolipin, a phospholipid found in cell membranes. These antibodies are associated with a high risk of miscarriage, particularly in women with APS. The mechanism by which aCL contributes to RSA involves both thrombosis and inflammation [115,116]. aCL promotes a prothrombotic state by activating platelets and endothelial cells, leading to clot formation in the placental vasculature, which can compromise blood flow to the fetus. Additionally, aCL can trigger an inflammatory response within the placenta, further contributing to pregnancy loss. Treatment strategies for women with RSA and aCL typically involve anticoagulation therapy, which has been shown to reduce the risk of miscarriage [117].

## 6. Clinical Recommendations

As the involvement of various immune cells in RSA is well-established, immune phenotyping can help guide targeted immunotherapies in RSA patients. For example, immune phenotyping of T cells in RSA can reveal an increased proportion of Th1 cells or elevated levels of Th1 cytokines. Similarly, cytokine imbalance and NK cell cytotoxicity can help with targeted immunotherapies such as intravenous immunoglobulin (IVIG), TNF inhibitors, or steroids. Studies have shown that IVIG can significantly increase the live birth rates in RSA patients [118]. There is also evidence that shows that treatment with IVIG can suppress the expression of Th1 cytokines promote Th2 cytokines and restore Th1/Th2 balance which is crucial for pregnancy maintenance [119]. Ahmadi et al. also showed that IVIG can increase the Th2 lymphocytes in patients with RSA [120]. Given the association of elevated TNF-α levels with RSA, the use of TNF-α inhibitors, such as etanercept or infliximab, has been explored as a therapeutic strategy [121]. Fu et al., in their study also reported that etanercept led to a significant decrease in TNF-α and NK cell activity compared to control groups [122]. Glucocorticoids, such as prednisolone, have immunosuppressive properties and can be used to dampen excessive immune activation, particularly NK cells in RSA [123]. There is sufficient analysis that has shown the efficacy of glucocorticoids in improving pregnancy rates and live birth rates [124]. In addition to IVIG, TNF-α inhibitors, and steroids, other immunomodulatory approaches, such as lymphocyte immunotherapy, low-dose aspirin, and heparin, have been investigated for the treatment of RSA. Immune phenotyping can aid in selecting appropriate candidates for these therapies based on the specific immune profile of the patient. For example, low-dose aspirin and heparin are often used in patients with antiphospholipid syndrome [125].

## 7. Conclusions

The immunological evaluation of patients with recurrent abortion is crucial for understanding the complex interplay of immune factors that contribute to RSA. A comprehensive assessment of immune cell populations, cytokine levels, and autoantibodies can provide valuable insights into potential underlying causes and guide therapeutic interventions. Ongoing research is essential to further elucidate the mechanisms of immune dysfunction in RSA and to develop targeted treatments that improve pregnancy outcomes for affected women.

## Figures and Tables

**Table 1:** The role of KIR in women with RSA.

Authors	Year	Study Design	Study Population	Methodology	Results	Outcomes
Akbari et al. [61]	2018	Case-control study	100 couples; RSA women	KIR genes and HLA allotypes with PCR-SSP for genotyping	Patients with RSA had elevated levels of *KIR2DS1* and paternal HLA-C2 compared to the control.	*KIR2DS1* and paternal HLA-C2 are risk factors for RSA
Mansour et al. [56]	2020	Case control	75 RSA women; 65 controls	17 KIR genes and HLA-C1 and HLA-C2 allotypes by PCR-SSP	KIR2DS2 and KIR2DL5A levels were lower in RSA compared to controls (*p* < 0.001)	KIR genes of the B haplotype can predict successful pregnancy.
Maftei et al. [62]	2023	Prospective study	108 RSA or women with recurrent implantation failure (RIF) (RSA: 30, RIF:78)	KIR AA haplotype with PCR-SSP	Patients with a KIR AA haplotype who underwent IVF were more likely to have a miscarriage compared to spontaneous pregnancy (*p* = 0.032).	KIR haplotype assessment can help predict the risk of RSA.
Alecsandru et al. [59]	2020	Prospective observational cohort study	204 RSA or RIF females	KIR and HLA-C genotypes assessed. HLA-C genes for all partners.	Higher rates of miscarriage were reported in KIR AA patients compared to KIR AB, and KIR BB. Birth rates significantly declined as HLA-C2 load increased in KIR AA women.	Increase in HLA-C levels in KIR AA women leads to poor outcomes for pregnancy.
Akbari et al. [55]	2020	Meta-analysis	11 studies	Web of Science, PubMed, Scopus, Google Scholar	*KIR3DL1* is a significant protective factor for RSA (*p* = 0.044) and KIR2DS2 and KIR2DS3 were risk factors for RSA.	Inhibitory KIR are protective in RSA and activating KIR are risk factors.
Ataei et al. [54]	2021	Case control	80; 40 with RSA and 40 normal pregnant women	Genotypes of KIR genes assessed and KIR3DL1 genotype frequency compared	KIR3DL1 levels are lower in RSA group compared to healthy controls (*p* = 0.012).	KIR3DL1 inhibitory genotype is protective in RSA.
Su et al. [58]	2018	Case control	110 RSA women, and 105 healthy controls	Genotypes of KIR genes and HLA-C assessed	KIR3DL1 levels were significantly lower in RSA whereas BB haplotype were significantly higher compared to control group.	RSA patients have reduced inhibitory genes and increased activating KIR genes.
Alharbi et al. [57]	2022	Case control	199 women; 69 RSA, 65 polycystic ovarian syndrome (PCO), 65 healthy	KIR genes and HLA allotypes with PCR-SSP for genotyping	KIR3DL1, 2DS4ins, 2DL2, and KIR2DS2 levels were significantly low in RSA compared to controls (*p* < 0.01).	KIR2DL1 with HLA-C2 is risk factor for RSA.
Elbaşı et al. [63]	2020	Case control	25 couples	KIR genes and HLA allotypes with PCR-SSP for genotyping	The KIR2DL5 levels were higher in both partners in RSA whereas KIR2DS3 levels were reduced in male RSA partners (*p* = 0.03).	Male HLA-C2 and female HLA-C1 ligand KIR receptors can affect the outcome of pregnancy.
Dambaeva et al. [64]	2016	Prospective study	139 RSA women	Genomic DNA was extracted using QuickGene DNA	RSA patients with KIR2DS1 had a high frequency of HLA-C2 (45.3%).	KIR2DS1 with HLA-C2 is associated with RSA.

PCR-SSP: Polymerase chain reaction with sequence-specific primers.

## References

[1] Deng T., Liao X., Zhu S. (2022). Recent Advances in Treatment of Recurrent Spontaneous Abortion. Obstet. Gynecol. Surv..

[2] Bender Atik R., Christiansen O.B., Elson J., Kolte A.M., Lewis S., Middeldorp S., Nelen W., Peramo B., Quenby S., Vermeulen N. (2018). ESHRE guideline: Recurrent pregnancy loss. Hum. Reprod. Open.

[3] (2012). Evaluation and treatment of recurrent pregnancy loss: A committee opinion. Fertil. Steril..

[4] Luu T., AlSubki L., Wolf K., Thees A., Ganieva U., Dambaeva S., Beaman K., Kwak-Kim J. (2022). Natural killer cell-mediated immunopathology in recurrent pregnancy losses. Explor. Immunol..

[5] Abdollahi E., Tavasolian F., Ghasemi N., Mirghanizadeh S.A., Azizi M., Ghoryani M., Samadi M. (2015). Association between lower frequency of R381Q variant (rs11209026) in IL-23 receptor gene and increased risk of recurrent spontaneous abortion (RSA). J. Immunotoxicol..

[6] Madani J., Aghebati-Maleki L., Gharibeh N., Pourakbari R., Yousefi M. (2022). Fetus, as an allograft, evades the maternal immunity. Transpl. Immunol..

[7] Maxwell A.J., You Y., Aldo P.B., Zhang Y., Ding J., Mor G. (2021). The role of the immune system during pregnancy: General concepts. Reproductive Immunology.

[8] Andreescu M. (2023). The impact of the use of immunosuppressive treatment after an embryo transfer in increasing the rate of live birth. Front. Med..

[9] Chakraborty D., Rumi M.A.K., Konno T., Soares M.J. (2011). Natural killer cells direct hemochorial placentation by regulating hypoxia-inducible factor dependent trophoblast lineage decisions. Proc. Natl. Acad. Sci. USA.

[10] Kim S.-G., Paek M.-Y., Ko I.G. (2019). Peripheral Blood Level of Natural Killer Cells in Pregnant Women with Recurrent Spontaneous Abortion during the 6–12 Weeks Gestation. Arch. Med. Health Sci..

[11] Mor G., Cardenas I. (2010). The immune system in pregnancy: A unique complexity. Am. J. Reprod. Immunol..

[12] Andreescu M., Frîncu F., Plotogea M., Mehedințu C. (2023). Recurrent Abortion and the Involvement of Killer-Cell Immunoglobulin-like Receptor (KIR) Genes, Activated T Cells, NK Abnormalities, and Cytokine Profiles. J. Clin. Med..

[13] Yang F., Zheng Q., Jin L. (2019). Dynamic Function and Composition Changes of Immune Cells During Normal and Pathological Pregnancy at the Maternal-Fetal Interface. Front. Immunol..

[14] Aldo P.B., Racicot K., Craviero V., Guller S., Romero R., Mor G. (2014). Trophoblast induces monocyte differentiation into CD14+/CD16+ macrophages. Am. J. Reprod. Immunol..

[15] Svensson-Arvelund J., Mehta R.B., Lindau R., Mirrasekhian E., Rodriguez-Martinez H., Berg G., Lash G.E., Jenmalm M.C., Ernerudh J. (2015). The human fetal placenta promotes tolerance against the semiallogeneic fetus by inducing regulatory T cells and homeostatic M2 macrophages. J. Immunol..

[16] Zhang X., Wei H. (2021). Role of Decidual Natural Killer Cells in Human Pregnancy and Related Pregnancy Complications. Front. Immunol..

[17] Male V., Moffett A. (2023). Natural Killer Cells in the Human Uterine Mucosa. Annu. Rev. Immunol..

[18] Vento-Tormo R., Efremova M., Botting R.A., Turco M.Y., Vento-Tormo M., Meyer K.B., Park J.E., Stephenson E., Polański K., Goncalves A. (2018). Single-cell reconstruction of the early maternal-fetal interface in humans. Nature.

[19] Feyaerts D., Benner M., Comitini G., Shadmanfar W., van der Heijden O.W.H., Joosten I., van der Molen R.G. (2024). NK cell receptor profiling of endometrial and decidual NK cells reveals pregnancy-induced adaptations. Front. Immunol..

[20] Fu B., Li X., Sun R., Tong X., Ling B., Tian Z., Wei H. (2013). Natural killer cells promote immune tolerance by regulating inflammatory TH17 cells at the human maternal-fetal interface. Proc. Natl. Acad. Sci. USA.

[21] Mahajan D., Sharma N.R., Kancharla S., Kolli P., Tripathy A., Sharma A.K., Singh S., Kumar S., Mohanty A.K., Jena M.K. (2022). Role of Natural Killer Cells during Pregnancy and Related Complications. Biomolecules.

[22] Kieffer T.E., Faas M.M., Scherjon S.A., Prins J.R. (2017). Pregnancy persistently affects memory T cell populations. J. Reprod. Immunol..

[23] Kieffer T.E., Laskewitz A., Scherjon S.A., Faas M.M., Prins J.R. (2019). Memory T cells in pregnancy. Front. Immunol..

[24] Jafarpour R., Pashangzadeh S., Mehdizadeh S., Bayatipoor H., Shojaei Z., Motallebnezhad M. (2020). Functional significance of lymphocytes in pregnancy and lymphocyte immunotherapy in infertility: A comprehensive review and update. Int. Immunopharmacol..

[25] Mosmann T.R., Cherwinski H., Bond M.W., Giedlin M.A., Coffman R.L. (1986). Two types of murine helper T cell clone. I. Definition according to profiles of lymphokine activities and secreted proteins. J. Immunol..

[26] Monteiro C., Kasahara T.M., Castro J.R., Sacramento P.M., Hygino J., Centurião N., Cassano T., Lopes L.M.F., Leite S., Gupta S. (2017). Pregnancy favors the expansion of circulating functional follicular helper T Cells. J. Reprod. Immunol..

[27] Logiodice F., Lombardelli L., Kullolli O., Haller H., Maggi E., Rukavina D., Piccinni M.-P. (2019). Decidual interleukin-22-producing CD4+ T cells (Th17/Th0/IL-22+ and Th17/Th2/IL-22+, Th2/IL-22+, Th0/IL-22+), which also produce IL-4, are involved in the success of pregnancy. Int. J. Mol. Sci..

[28] Billingham R.E., Brent L., Medawar P.B. (2010). ‘Actively acquired tolerance’ of foreign cells. 1953. J. Immunol.

[29] Germain S.J., Sacks G.P., Sooranna S.R., Sargent I.L., Redman C.W. (2007). Systemic inflammatory priming in normal pregnancy and preeclampsia: The role of circulating syncytiotrophoblast microparticles. J. Immunol..

[30] Graham J.J., Longhi M.S., Heneghan M.A. (2021). T helper cell immunity in pregnancy and influence on autoimmune disease progression. J. Autoimmun..

[31] Kim B., Kim T.H. (2018). Fundamental role of dendritic cells in inducing Th2 responses. Korean J. Intern. Med..

[32] Wang J., Han T., Zhu X. (2024). Role of maternal-fetal immune tolerance in the establishment and maintenance of pregnancy. Chin. Med J..

[33] Chatterjee P., Chiasson V.L., Bounds K.R., Mitchell B.M. (2014). Regulation of the Anti-Inflammatory Cytokines Interleukin-4 and Interleukin-10 during Pregnancy. Front. Immunol..

[34] Iwaszko M., Biały S., Bogunia-Kubik K. (2021). Significance of Interleukin (IL)-4 and IL-13 in Inflammatory Arthritis. Cells.

[35] Holtan S.G., Chen Y., Kaimal R., Creedon D.J., Enninga E.A.L., Nevala W.K., Markovic S.N. (2015). Growth modeling of the maternal cytokine milieu throughout normal pregnancy: Macrophage-derived chemokine decreases as inflammation/counterregulation increases. J. Immunol. Res..

[36] Zare M., Jahromi B.N., Gharesi-Fard B. (2019). Analysis of the frequencies and functions of CD4+ CD25+ CD127low/neg, CD4+ HLA-G+, and CD8+ HLA-G+ regulatory T cells in pre-eclampsia. J. Reprod. Immunol..

[37] Krop J., Heidt S., Claas F.H.J., Eikmans M. (2020). Regulatory T Cells in Pregnancy: It Is Not All About FoxP3. Front. Immunol..

[38] Zhang Y.-H., Sun H.-X. (2020). Immune checkpoint molecules in pregnancy: Focus on regulatory T cells. Eur. J. Immunol..

[39] Samstein R.M., Josefowicz S.Z., Arvey A., Treuting P.M., Rudensky A.Y. (2012). Extrathymic generation of regulatory T cells in placental mammals mitigates maternal-fetal conflict. Cell.

[40] Xin L., Ertelt J.M., Rowe J.H., Jiang T.T., Kinder J.M., Chaturvedi V., Elahi S., Way S.S. (2014). Cutting edge: Committed Th1 CD4+ T cell differentiation blocks pregnancy-induced Foxp3 expression with antigen-specific fetal loss. J. Immunol..

[41] Figueiredo A.S., Schumacher A. (2016). The T helper type 17/regulatory T cell paradigm in pregnancy. Immunology.

[42] Hosseini S., Shokri F., Ansari Pour S., Jeddi-Tehrani M., Nikoo S., Yousefi M., Zarnani A.-H. (2016). A shift in the balance of T17 and Treg cells in menstrual blood of women with unexplained recurrent spontaneous abortion. J. Reprod. Immunol..

[43] Pongcharoen S., Supalap K. (2009). Interleukin-17 increased progesterone secretion by JEG-3 human choriocarcinoma cells. Am. J. Reprod. Immunol..

[44] Luu T.V., Thees A., Ganieva U., Dambaeva S., Beaman K., Kwak-Kim J. (2022). T regulatory, Th17, and treg/Th17 ratio, in pregnant women with recurrent pregnancy losses and normal pregnant women. Fertil. Steril..

[45] Nakashima A., Ito M., Yoneda S., Shiozaki A., Hidaka T., Saito S. (2010). Circulating and decidual Th17 cell levels in healthy pregnancy. Am. J. Reprod. Immunol..

[46] Tang C., Hu W. (2023). The role of Th17 and Treg cells in normal pregnancy and unexplained recurrent spontaneous abortion (URSA): New insights into immune mechanisms. Placenta.

[47] Braga A., Neves E., Guimarães J., Braga J., Vasconcelos C. (2022). Th17/Regulatory T cells ratio evolution: A prospective study in a group of healthy pregnant women. J. Reprod. Immunol..

[48] Romanowska-Próchnicka K., Felis-Giemza A., Olesińska M., Wojdasiewicz P., Paradowska-Gorycka A., Szukiewicz D. (2021). The Role of TNF-α and Anti-TNF-α Agents during Preconception, Pregnancy, and Breastfeeding. Int. J. Mol. Sci..

[49] Wen B., Liao H., Lin W., Li Z., Ma X., Xu Q., Yu F. (2023). The Role of TGF-β during Pregnancy and Pregnancy Complications. Int. J. Mol. Sci..

[50] Hadinedoushan H., Mirahmadian M., Aflatounian A. (2007). Increased natural killer cell cytotoxicity and IL-2 production in recurrent spontaneous abortion. Am. J. Reprod. Immunol..

[51] Karami N., Boroujerdnia M.G., Nikbakht R., Khodadadi A. (2012). Enhancement of peripheral blood CD56(dim) cell and NK cell cytotoxicity in women with recurrent spontaneous abortion or in vitro fertilization failure. J. Reprod. Immunol..

[52] Shakhar K., Ben-Eliyahu S., Loewenthal R., Rosenne E., Carp H. (2003). Differences in number and activity of peripheral natural killer cells in primary versus secondary recurrent miscarriage. Fertil. Steril..

[53] Papúchová H., Meissner T.B., Li Q., Strominger J.L., Tilburgs T. (2019). The Dual Role of HLA-C in Tolerance and Immunity at the Maternal-Fetal Interface. Front. Immunol..

[54] Ataei M., Mirzaei M., Inanloo F., Maleki N., Rad S.S., Noorbakhsh S.M., Atousa K. (2021). KIR3DL1 gene genotype in patients with spontaneous recurrent abortion. Arch. Venez. De Farmacol. Y Ter..

[55] Akbari S., Shahsavar F., Karami R., Yari F., Anbari K., Ahmadi S.A.Y. (2020). Recurrent Spontaneous Abortion (RSA) and Maternal KIR Genes: A Comprehensive Meta-Analysis. JBRA Assist Reprod.

[56] Mansour L., Alkhuriji A., Babay Z.A., Alqadheeb S., Al-Khulaifi F., Al-Talhi R., Alomar S. (2020). Association of Killer Immunoglobulin-Like Receptor and Human Leukocyte Antigen Class I Ligand with Recurrent Abortion in Saudi Women. Genet. Test. Mol. Biomark..

[57] Alharbi H.M., Alkhuriji A.F., Alomar S.Y., Babay Z.A., Alnafjan A.A., Alobaid H.M., Allharbi W.G., Mansour L.A. (2022). Association of recurrent spontaneous abortion with polycystic ovarian syndrome under the influence of killer immunoglobulin like receptors. J. King Saud Univ. Sci..

[58] Su N., Wang H., Zhang B., Kang Y., Guo Q., Xiao H., Yang H., Liao S. (2018). Maternal natural killer cell immunoglobulin receptor genes and human leukocyte antigen-C ligands influence recurrent spontaneous abortion in the Han Chinese population. Exp. Ther. Med..

[59] Alecsandru D., Barrio A., Garrido N., Aparicio P., Pellicer A., Moffett A., García-Velasco J.A. (2020). Parental human leukocyte antigen-C allotypes are predictive of live birth rate and risk of poor placentation in assisted reproductive treatment. Fertil. Steril..

[60] Yang X., Meng T. (2021). Killer-cell immunoglobulin-like receptor/human leukocyte antigen-C combination and ‘great obstetrical syndromes’ (Review). Exp. Ther. Med..

[61] Akbari S., Ahmadi S.A.Y., Shahsavar F. (2018). The relationship of maternal KIR and parental HLA-C genes with risk of recurrent spontaneous abortion: A regional study in Lorestan province, Iran. Crescent J. Med. Biol. Sci..

[62] Maftei R., Doroftei B., Popa R., Harabor V., Adam A.-M., Popa C., Harabor A., Adam G., Nechita A., Vasilache I.-A. (2023). The Influence of Maternal KIR Haplotype on the Reproductive Outcomes after Single Embryo Transfer in IVF Cycles in Patients with Recurrent Pregnancy Loss and Implantation Failure—A Single Center Experience. J. Clin. Med..

[63] Elbaşı M.O., Tulunay A., Karagözoğlu H., Kahraman S., Ekşioğlu-Demiralp E. (2020). Maternal killer-cell immunoglobulin-like receptors and paternal human leukocyte antigen ligands in recurrent pregnancy loss cases in Turkey. Clin. Exp. Reprod. Med..

[64] Dambaeva S.V., Lee D.H., Sung N., Chen C.Y., Bao S., Gilman-Sachs A., Kwak-Kim J., Beaman K.D. (2016). Recurrent Pregnancy Loss in Women with Killer Cell Immunoglobulin-Like Receptor KIR2DS1 is Associated with an Increased HLA-C2 Allelic Frequency. Am. J. Reprod. Immunol..

[65] Keller C.C., Eikmans M., van der Hoorn M.P., Lashley L. (2020). Recurrent miscarriages and the association with regulatory T cells; A systematic review. J. Reprod. Immunol..

[66] Luo L., Zeng X., Huang Z., Luo S., Qin L., Li S. (2020). Reduced frequency and functional defects of CD4+CD25highCD127low/− regulatory T cells in patients with unexplained recurrent spontaneous abortion. Reprod. Biol. Endocrinol..

[67] Winger E.E., Reed J.L. (2011). Low circulating CD4(+) CD25(+) Foxp3(+) T regulatory cell levels predict miscarriage risk in newly pregnant women with a history of failure. Am. J. Reprod. Immunol..

[68] Care A.S., Bourque S.L., Morton J.S., Hjartarson E.P., Robertson S.A., Davidge S.T. (2018). Reduction in Regulatory T Cells in Early Pregnancy Causes Uterine Artery Dysfunction in Mice. Hypertension.

[69] Farshchi M., Abdollahi E., Saghafi N., Hosseini A., Fallahi S., Rostami S., Rostami P., Rafatpanah H., Habibagahi M. (2022). Evaluation of Th17 and Treg cytokines in patients with unexplained recurrent pregnancy loss. J. Clin. Transl. Res..

[70] Yang X., Tian Y., Zheng L., Luu T., Kwak-Kim J. (2023). The Update Immune-Regulatory Role of Pro- and Anti-Inflammatory Cytokines in Recurrent Pregnancy Losses. Int. J. Mol. Sci..

[71] Lee S., Kim J., Hur S., Kim C., Na B., Lee M., Gilman-Sachs A., Kwak-Kim J. (2011). An imbalance in interleukin-17-producing T and Foxp3+ regulatory T cells in women with idiopathic recurrent pregnancy loss. Hum. Reprod..

[72] Wang W., Sung N., Gilman-Sachs A., Kwak-Kim J. (2020). T helper (Th) cell profiles in pregnancy and recurrent pregnancy losses: Th1/Th2/Th9/Th17/Th22/Tfh cells. Front. Immunol..

[73] Kang Y., Xie Q., Chen S., Li Q., Dong X., Zhang T., Fu S., Lei Q., Huang D. (2023). Research progress of immune balance and genetic polymorphism in unexplained recurrent abortion. Explor. Immunol..

[74] Gao Y., Wang P. (2015). Increased CD56 (+) NK cells and enhanced Th1 responses in human unexplained recurrent spontaneous abortion. Genet. Mol. Res..

[75] Jena M.K., Nayak N., Chen K., Nayak N.R. (2019). Role of macrophages in pregnancy and related complications. Arch. Immunol. Et Ther. Exp..

[76] Ding J., Yin T., Yan N., Cheng Y., Yang J. (2019). FasL on decidual macrophages mediates trophoblast apoptosis: A potential cause of recurrent miscarriage. Int. J. Mol. Med..

[77] Wang H., He M., Hou Y., Chen S., Zhang X., Zhang M., Ji X. (2016). Role of decidual CD14+ macrophages in the homeostasis of maternal–fetal interface and the differentiation capacity of the cells during pregnancy and parturition. Placenta.

[78] Zhao Q.-Y., Li Q.-H., Fu Y.-Y., Ren C.-E., Jiang A.-F., Meng Y.-H. (2022). Decidual macrophages in recurrent spontaneous abortion. Front. Immunol..

[79] Pérez S., Rius-Pérez S. (2022). Macrophage Polarization and Reprogramming in Acute Inflammation: A Redox Perspective. Antioxidants.

[80] Biswas S.K., Mantovani A. (2010). Macrophage plasticity and interaction with lymphocyte subsets: Cancer as a paradigm. Nat. Immunol..

[81] Yunna C., Mengru H., Lei W., Weidong C. (2020). Macrophage M1/M2 polarization. Eur. J. Pharmacol..

[82] Jaiswal M.K., Mallers T.M., Larsen B., Kwak-Kim J., Chaouat G., Gilman-Sachs A., Beaman K.D. (2012). V-ATPase upregulation during early pregnancy: A possible link to establishment of an inflammatory response during preimplantation period of pregnancy. Reproduction.

[83] Hamilton S., Oomomian Y., Stephen G., Shynlova O., Tower C.L., Garrod A., Lye S.J., Jones R.L. (2012). Macrophages infiltrate the human and rat decidua during term and preterm labor: Evidence that decidual inflammation precedes labor. Biol. Reprod..

[84] Patel U., Rajasingh S., Samanta S., Cao T., Dawn B., Rajasingh J. (2017). Macrophage polarization in response to epigenetic modifiers during infection and inflammation. Drug Discov. Today.

[85] Mantovani A., Sica A., Sozzani S., Allavena P., Vecchi A., Locati M. (2004). The chemokine system in diverse forms of macrophage activation and polarization. Trends Immunol..

[86] Lampiasi N., Russo R., Zito F. (2016). The Alternative Faces of Macrophage Generate Osteoclasts. Biomed Res. Int..

[87] Tsao F.-Y., Wu M.-Y., Chang Y.-L., Wu C.-T., Ho H.-N. (2018). M1 macrophages decrease in the deciduae from normal pregnancies but not from spontaneous abortions or unexplained recurrent spontaneous abortions. J. Formos. Med. Assoc..

[88] Liu X., Jiang M., Ren L., Zhang A., Zhao M., Zhang H., Jiang Y., Hu X. (2018). Decidual macrophage M1 polarization contributes to adverse pregnancy induced by Toxoplasma gondii PRU strain infection. Microb. Pathog..

[89] Brown M.B., von Chamier M., Allam A.B., Reyes L. (2014). M1/M2 macrophage polarity in normal and complicated pregnancy. Front. Immunol..

[90] Ding J., Zhang Y., Cai X., Zhang Y., Yan S., Wang J., Zhang S., Yin T., Yang C., Yang J. (2021). Extracellular vesicles derived from M1 macrophages deliver miR-146a-5p and miR-146b-5p to suppress trophoblast migration and invasion by targeting TRAF6 in recurrent spontaneous abortion. Theranostics.

[91] Darmochwal-Kolarz D., Rolinski J., Tabarkiewicz J., Leszczynska-Gorzelak B., Buczkowski J., Wojas K., Oleszczuk J. (2003). Myeloid and lymphoid dendritic cells in normal pregnancy and pre-eclampsia. Clin. Exp. Immunol..

[92] Qian Z.-D., Huang L.-L., Zhu X.-M. (2015). An immunohistochemical study of CD83- and CD1a-positive dendritic cells in the decidua of women with recurrent spontaneous abortion. Eur. J. Med. Res..

[93] Liu J., Dong P., Jia N., Wen X., Luo L., Wang S., Li J. (2022). The expression of intracellular cytokines of decidual natural killer cells in unexplained recurrent pregnancy loss. J. Matern. Fetal Neonatal Med..

[94] Brogin Moreli J., Cirino Ruocco A.M., Vernini J.M., Rudge M.V., Calderon I.M. (2012). Interleukin 10 and tumor necrosis factor-alpha in pregnancy: Aspects of interest in clinical obstetrics. ISRN Obstet. Gynecol.

[95] Dong P., Wen X., Liu J., Yan C.Y., Yuan J., Luo L.R., Hu Q.F., Li J. (2017). Simultaneous detection of decidual Th1/Th2 and NK1/NK2 immunophenotyping in unknown recurrent miscarriage using 8-color flow cytometry with FSC/Vt extended strategy. Biosci. Rep..

[96] Calleja-Agius J., Jauniaux E., Pizzey A.R., Muttukrishna S. (2012). Investigation of systemic inflammatory response in first trimester pregnancy failure. Hum. Reprod..

[97] Li M.-M., Lin J., Wu H.-F., Zheng G.-J., Cai R.-N. (2023). Analysis of the risk factors in patients with unexplained recurrent spontaneous abortion. Am. J. Reprod. Immunol..

[98] Alkhuriji A.F., Al Omar S.Y., Babay Z.A., El-khadragy M.F., Mansour L.A., Alharbi W.G., Khalil M.I. (2020). Association of IL-1β, IL-6, TNF-α, and TGFβ1 Gene Polymorphisms with Recurrent Spontaneous Abortion in Polycystic Ovary Syndrome. Dis. Markers.

[99] Begum A., Mishra A., Das C.R., Das S., Dutta R., Kashyap N., Bose P.D., Bose S. (2021). Impact of TNF-α profile in recurrent pregnancy loss pathogenesis: A patient based study from Assam. J. Reprod. Immunol..

[100] Jiang Y., Zou Q., Zhang N., Chen J., Chen X., You Q., Wu H. (2022). Tumour necrosis factor inhibitor combined with intravenous immunoglobulin and heparin for treatment of recurrent spontaneous abortion: A two-centre, retrospective, cohort study. J. Clin. Pharm. Ther..

[101] Piosik Z.M., Goegebeur Y., Klitkou L., Steffensen R., Christiansen O.B. (2013). Plasma TNF-α levels are higher in early pregnancy in patients with secondary compared with primary recurrent miscarriage. Am. J. Reprod. Immunol..

[102] Chen L.M., Liu B., Zhao H.B., Stone P., Chen Q., Chamley L. (2010). IL-6, TNFalpha and TGFbeta promote nonapoptotic trophoblast deportation and subsequently causes endothelial cell activation. Placenta.

[103] Zhu L., Chen H., Liu M., Yuan Y., Wang Z., Chen Y., Wei J., Su F., Zhang J. (2017). Treg/Th17 Cell Imbalance and IL-6 Profile in Patients with Unexplained Recurrent Spontaneous Abortion. Reprod. Sci..

[104] Thaker R., Oza H., Verma V., Gor M., Kumar S. (2021). The Association of Circulatory Cytokines (IL-6 and IL-10) Level with Spontaneous Abortion—A Preliminary Observation. Reprod. Sci..

[105] Liu Z., Sun S., Xu H., Zhang X., Chen C., Fu R., Li C., Guo F., Zhao A. (2022). Prognostic analysis of antibody typing and treatment for antiphospholipid syndrome-related recurrent spontaneous abortion. Int. J. Gynecol. Obstet..

[106] Beltagy A., Trespidi L., Gerosa M., Ossola M.W., Meroni P.L., Chighizola C.B. (2021). Anti-phospholipid antibodies and reproductive failures. Am. J. Reprod. Immunol..

[107] Yu X., He L. (2021). Aspirin and heparin in the treatment of recurrent spontaneous abortion associated with antiphospholipid antibody syndrome: A systematic review and meta-analysis. Exp. Ther. Med..

[108] Liu T., Guo X., Liao Y., Liu Y., Zhu Y., Chen X. (2022). Correlation Between the Presence of Antinuclear Antibodies and Recurrent Pregnancy Loss: A Mini Review. Front. Endocrinol..

[109] Shankarkumar U., Pradhan V.D., Patwardhan M.M., Shankarkumar A., Ghosh K. (2011). Autoantibody profile and other immunological parameters in recurrent spontaneous abortion patients. Niger. Med. J..

[110] Chen S., Yang G., Wu P., Sun Y., Dai F., He Y., Qian H., Liu Y., Shi G. (2020). Antinuclear antibodies positivity is a risk factor of recurrent pregnancy loss: A meta-analysis. Semin. Arthritis Rheum..

[111] Xie J., Jiang L., Sadhukhan A., Yang S., Yao Q., Zhou P., Rao J., Jin M. (2020). Effect of antithyroid antibodies on women with recurrent miscarriage: A meta-analysis. Am. J. Reprod. Immunol..

[112] Song H., Cui T., Shi S., Xiao H., Wei A. (2024). Effect of anti-thyroid antibodies on recurrent miscarriage: A meta-analysis. J. Obstet. Gynaecol. Res..

[113] Liu M., Wang D., Zhu L., Yin J., Ji X., Zhong Y., Gao Y., Zhang J., Liu Y., Zhang R. (2022). Association of thyroid peroxidase antibodies with the rate of first-trimester miscarriage in euthyroid women with unexplained recurrent spontaneous abortion. Front. Endocrinol..

[114] Miko E., Meggyes M., Doba K., Farkas N., Bogar B., Barakonyi A., Szereday L., Szekeres-Bartho J., Mezosi E. (2017). Characteristics of peripheral blood NK and NKT-like cells in euthyroid and subclinical hypothyroid women with thyroid autoimmunity experiencing reproductive failure. J. Reprod. Immunol..

[115] Yadav S., Chaurasia S., Mirza S., Resident Y.P. (2023). To evaluate the prevalence of anticardiolipin antibodies among women with recurrent abortions and to determine any relation between anticardiolipin antibodies and number of abortions and their gestational age of abortions. J. Cardiovasc. Dis. Res..

[116] Yokote R., Kuwabara Y., Kasano S., Yonezawa M., Ouchi N., Ichikawa T., Suzuki S., Takeshita T. (2023). Risk factors for persistent positive anticardiolipin antibodies in women with recurrent pregnancy loss. J. Reprod. Immunol..

[117] Shaikhomar O.A., Ali S.T. (2022). A Comparative Analysis of Anticardiolipin, Anti-Β2-Glycoprotein-1, and Lupus Anticoagulants in Saudi Women with Recurrent Spontaneous Abortions. J. Pers. Med..

[118] Shi Y., Tan D., Hao B., Zhang X., Geng W., Wang Y., Sun J., Zhao Y. (2022). Efficacy of intravenous immunoglobulin in the treatment of recurrent spontaneous abortion: A systematic review and meta-analysis. Am. J. Reprod. Immunol..

[119] Graphou O., Chioti A., Pantazi A., Tsukoura C., Kontopoulou V., Guorgiadou E., Balafoutas C., Koussoulakos S., Margaritis L.H., Varla-Leftherioti M. (2003). Effect of intravenous immunoglobulin treatment on the Th1/Th2 balance in women with recurrent spontaneous abortions. Am. J. Reprod. Immunol..

[120] Ahmadi M., Abdolmohammadi-vahid S., Ghaebi M., Aghebati-Maleki L., Afkham A., Danaii S., Abdollahi-Fard S., Heidari L., Jadidi-Niaragh F., Younesi V. (2017). Effect of Intravenous immunoglobulin on Th1 and Th2 lymphocytes and improvement of pregnancy outcome in recurrent pregnancy loss (RPL). Biomed. Pharmacother..

[121] Wu H., You Q., Jiang Y., Mu F. (2021). Tumor necrosis factor inhibitors as therapeutic agents for recurrent spontaneous abortion (Review). Mol. Med. Rep..

[122] Fu J., Li L., Qi L., Zhao L. (2019). A randomized controlled trial of etanercept in the treatment of refractory recurrent spontaneous abortion with innate immune disorders. Taiwan. J. Obstet. Gynecol..

[123] Roumandeh N., Zare A., Saremi A.T. (2018). Immunology of Recurrent Spontaneous Abortion. saremjm.

[124] Li T., Yuan Y., Liu H., Lu Q., Mu R. (2022). Glucocorticoids Improve the Pregnancy Rate and Outcome in Women with Unexplained Positive Autoantibodies: A Systematic Review and Meta-Analysis. Front. Med..

[125] Shi T., Gu Z.-D., Diao Q.-z. (2021). Meta-analysis on aspirin combined with low-molecular-weight heparin for improving the live birth rate in patients with antiphospholipid syndrome and its correlation with d-dimer levels. Medicine.

